# New Medical Journal

**Published:** 1872-11

**Authors:** 


NEW MEDICAL JOURNAL.—*Archives of Scientific and Practical Medicine.*—Dr. Brown-Sequard proposes soon to begin, with the assistance of his friend, Dr. E. Seguin, and several New York, Boston, and Philadelphia Physicians and Surgeons, the publication of a new Medical Journal, under the above title. Subscriptions will be paid or remitted to Messrs. J. B. Lippincott & Co,, No. 715 and 717 Market Street, Philadelphia, or No. 25 Bond Street, New York City. The annual subscription price will be Four Dollars, to be paid in full, in advance. The first number, bearing date January 1st, 1873, will appear before the end of December, 1872.

